# Oviposition Site-Selection by *Bactrocera dorsalis* Is Mediated through an Innate Recognition Template Tuned to γ-Octalactone

**DOI:** 10.1371/journal.pone.0085764

**Published:** 2014-01-23

**Authors:** Kamala Jayanthi Pagadala Damodaram, Vivek Kempraj, Ravindra Mahadappa Aurade, Ravindra Kothapalli Venkataramanappa, Bakthavatsalam Nandagopal, Abraham Verghese, Toby Bruce

**Affiliations:** 1 National Fellow Lab, Division of Entomology and Nematology, Indian Institute of Horticultural Research, Bangalore, India; 2 Department of Ecology, National Bureau of Agriculturally Important Insects, Bangalore, India; 3 Biological Chemistry and Crop Protection, Rothamsted Research, West Common, Harpenden, United Kingdom; AgroParisTech, France

## Abstract

Innate recognition templates (IRTs) in insects are developed through many years of evolution. Here we investigated olfactory cues mediating oviposition behavior in the oriental fruit fly, *Bactrocera dorsalis*, and their role in triggering an IRT for oviposition site recognition. Behavioral assays with electrophysiologically active compounds from a preferred host, mango, revealed that one of the volatiles tested, γ-octalactone, had a powerful effect in eliciting oviposition by gravid *B. dorsalis* females. Electrophysiological responses were obtained and flies clearly differentiated between treated and untreated substrates over a wide range of concentrations of γ-octalactone. It triggered an innate response in flies, overriding inputs from other modalities required for oviposition site evaluation. A complex blend of mango volatiles not containing γ-octalactone elicited low levels of oviposition, whereas γ-octalactone alone elicited more oviposition response. Naïve flies with different rearing histories showed similar responses to γ-octalactone. Taken together, these results indicate that oviposition site selection in *B. dorsalis* is mediated through an IRT tuned to γ-octalactone. Our study provides empirical data on a cue underpinning innate behavior and may also find use in control operations against this invasive horticultural pest.

## Introduction

Insects make vital decisions about selection of food, mates or oviposition sites through pre-constructed recognition templates [1], [2]. These templates can be innate or acquired through learning and experience [2]. Innate recognition templates (IRTs) are embedded into the genome of insects and are transferred genetically to offspring so that when they are exposed to a fixed cue, or a fixed set of cues, a particular behavior is elicited without having to be learnt. Such recognition templates in insects are thought to be numerous; however, there is a paucity of information about the cues that trigger them. IRTs are triggered by external stimuli that occur within the context of the insect's ecology.

An example of a behavior that may be directed through an IRT is oviposition site-selection by insects [3], [4]. A predilection for favorable oviposition sites by adult insects is essential for successful development and fitness of their progeny [5]. Owing to strong competition for oviposition sites and processing time constraints, a female insect has to rapidly evaluate and direct her eggs into suitable oviposition sites [6]. This process of evaluation and egg-laying is instigated only while the insect is gravid. Oviposition site-selection in insects is assisted by a plethora of site-specific cues [7]–[9], but insects have to choose the most reliable and specific cues to override noise and channel appropriate sensory information efficiently during this crucial process. In insects, learning of certain crucial behaviors is impossible due to their short life span and cost incurred during learning. Therefore, crucial behaviors become innate, are embedded into the genome, and passed on by the parents to offspring as IRTs [10]–[12]. Through co-evolution with their hosts, insects construct recognition templates to crucial cues that aid in faster processing of information in their brain [1]–[3]. However, the oviposition-stimulants to which insects have developed IRTs remain elusive [13].

Insect olfactory driven behaviors can have an “innate bias” towards certain stimuli or be learned by association [14]. When innate bias occurs, the insects respond to the critical stimulus, rather than the other environmental stimuli; it is possible that the insect can be tricked by these cues even if they are outside their usual context. Here we studied the oviposition behavior of the oriental fruit fly, *Bactrocera dorsalis* (Diptera: Tephritidae), in relation to exposure to volatile semiochemicals from ripe mango (cv. Alphonso) that are known to be electrophysiologically active for this insect [15], [16]. We found that oviposition site-selection depends almost entirely on one of these volatile compounds, γ-octalactone. Upon careful experimentation, involving insects with different rearing histories, it was observed that oviposition behavior in *B. dorsalis* is mediated through an IRT tuned to γ-octalactone. Discovering oviposition-stimulants triggering an IRT will help us ‘trick’ gravid *B. dorsalis* females, a devastating invasive pest of horticultural importance [17]–[19], into traps.

## Materials and Methods

### Insects

*B. dorsalis* were reared and maintained at the Division of Entomology and Nematology, Indian Institute of Horticultural Research, Bangalore, India. Flies reared on a standard diet of mangoes or guavas were used for all experiments except the one in which rearing history was compared. For this, three populations were reared individually on mango, guava or banana for 3 generations, with one-generation cycle of 1 month. Fruits were exposed to gravid females for oviposition. Oviposited fruits were placed on fine sterilized sand to aid larval development and pupation. Pupae were separated by sieving the sand and placed in screened cages (30×30×30 cm) for the emergence of adults. Adult flies that emerged were provided with yeast, sugar and moistened cotton swabs *ad libitum*. Adults, 7-days old, were allowed to mate and gravid females (15-days old) were separated into another cage for behavioral assays. All colonies were maintained at optimum growth conditions of 28±2°C, 75% RH and a photoperiod of 12-h light/12-h dark cycles. For all experiments newly gravid females (30 flies) were used.

### Chemicals

All chemicals were procured from Sigma-Aldrich, St. Louis, USA and were of >95% purity.

### Screening for oviposition stimulants

We previously identified 7 electrophysiologically active headspace volatiles from mango cv. Alphonso, which is a highly preferred host plant for *B. dorsalis*, and showed that they function as attractants enabling flies to locate host fruit [16]. In order to test the hypothesis that these compounds could function as oviposition-stimulants, we conducted oviposition behavior bioassays in the current study. Thus, electrophysiologically active volatiles on filter paper discs (5 µL of 0.05 ppm; 50 mm disc diameter) were presented to gravid female fruit flies singly. Chemical standards of previously identified electrophysiologically active headspace volatile cues from host fruit, mango (cv. Alphonso) [16], namely, heptane, myrcene, (*Z*)-ocimene, (*E*)-ocimene, allo-ocimene, (*Z*)-myroxide and γ-octalactone were screened for their oviposition stimulant activity. Briefly, fresh gravid female flies (30 flies) were released into screened cages. They were allowed to acclimatize for 3-h prior to screening. Singly, each selected headspace volatile (5 µL of 0.05 ppm) was applied on 50 mm filter paper discs and presented to the flies. Hallmark behaviors of oviposition stimulation such as ovipositor extension and puncturing action due to excitation were observed and recorded. γ-Octalactone was the only headspace volatile that elicited oviposition behavior and was selected for further studies. The screening for each cue was conducted in triplicates (See Video S1 for oviposition behavior).

### Binary-Choice Oviposition and Single Plate Two-Choice Assay

Pulp discs were used as oviposition substrate to determine the efficiency of oviposition-stimulants in attracting and instigating oviposition. Pulp discs were prepared by mixing water (100 ml), mango pulp (cv. Totapuri; 50 g) and agarose (0.8 g). The pulp of cv. Totapuri was used in making pulp disc, as it did not contain the identified oviposition stimulant, γ-octalactone. The mixture was autoclaved at 121°C (15 psi) for 15 min. The molten mixture was cooled to 40°C and appropriate amount of the identified oviposition stimulant, γ-octalactone was added. This mixture was poured into circular molds precooled to −20°C. This was done to enhance setting of the mixture and lower volatile vaporization. The volatile emission rates from pulp discs were determined by collecting headspace volatiles from pulp discs placed in glass container through which charcoal filtered air was drawn using a system of pumps and passed through Porapak Q (mesh: 50–80; 50 mg/column) filters that trapped the volatiles as air exited the container. The volatiles trapped in Porapak Q filters were extracted by passing redistilled di-ethyl ether (750 µl) through the filters. Volatiles were then analyzed using a Hewlett-Packard GC system equipped with a HP-1 column (0.25 µm ID). Ten microliter of the extract was used for GC analysis. Pulp discs were prepared freshly when required. Pulp discs without γ-octalactone were used as control.

For binary-choice oviposition assay, pulp discs containing the identified oviposition stimulant, γ-octalactone and a control disc were presented to gravid females (15-day aged, 30 flies) for 24 h. The number of eggs laid in the test and control was enumerated by dissecting the pulp discs under a stereomicroscope (Leica, Germany). Twenty replicates for each volatile compound were conducted. For single plate two-choice assay, two choice plates were prepared by mixing appropriate amount of the identified oviposition stimulant, γ-octalactone or EAD active mango (cv. Alphonso) headspace volatiles into molten pulp mix or only agarose at 40°C. Two-choice dish was made by dividing a 90-mm petri dish (Tarsons, India) with a razor blade and pouring 2 samples of oviposition substrate into each half. Each plate was therefore divided into 2 halves with: (i) one half of the oviposition substrate/agarose containing EAD active volatiles (see *Screening of oviposition stimulants* in *Materials and Methods* section for details) or γ-octalactone at natural concentration and (ii) another half of the oviposition substrate/agarose without the identified oviposition stimulant; according to experimental needs (see *Results* for details). The razor blade was removed after the substrate hardened (20 min). For each test, 30 gravid females were allowed to sample the plates in a cage for 24 h. The oviposition preference was determined by counting the amount of eggs on each half of the 2 choice plates.

### Preference and electrophysiology studies

EAG (Syntech, Netherlands) recordings were done with Ag-AgCl_2_ glass microelectrodes filled with saline solution (50 mM NaCl or 50 mM KCl). During recording, gravid female flies were anaesthetized by chilling and their whole antennae were removed with a pair of micro-scissors for EAG preparations. The antenna was placed between the electrodes and the signals passed were detected by a high impedance amplifier (IDAC 2) and analyzed using EAG software supplied with the instrument. The recordings were taken by pulsing air (1000 millisecond) containing the identified cue diluted in hexane to produce different concentrations ranging from 0.05 to 100 ppm over the antenna. Newly prepared antenna was used for each recording. Six recordings per concentration were done and the antennal responses in mV were recorded and a dose-response curve was plotted. Oviposition substrate was prepared as described in the binary-choice assay method. The molten substrate containing appropriate concentrations of oviposition stimulant was poured into molds. After setting of the pulp discs containing different test concentrations of γ-octalactone ranging from 0.05 to 100 ppm, the discs were placed in separate cages with gravid females (15-day aged, 30 flies) for 24 h during which oviposition occurred.

## Results

### γ-Octalactone instigates oviposition in *B. dorsalis*

**Figure 1:**
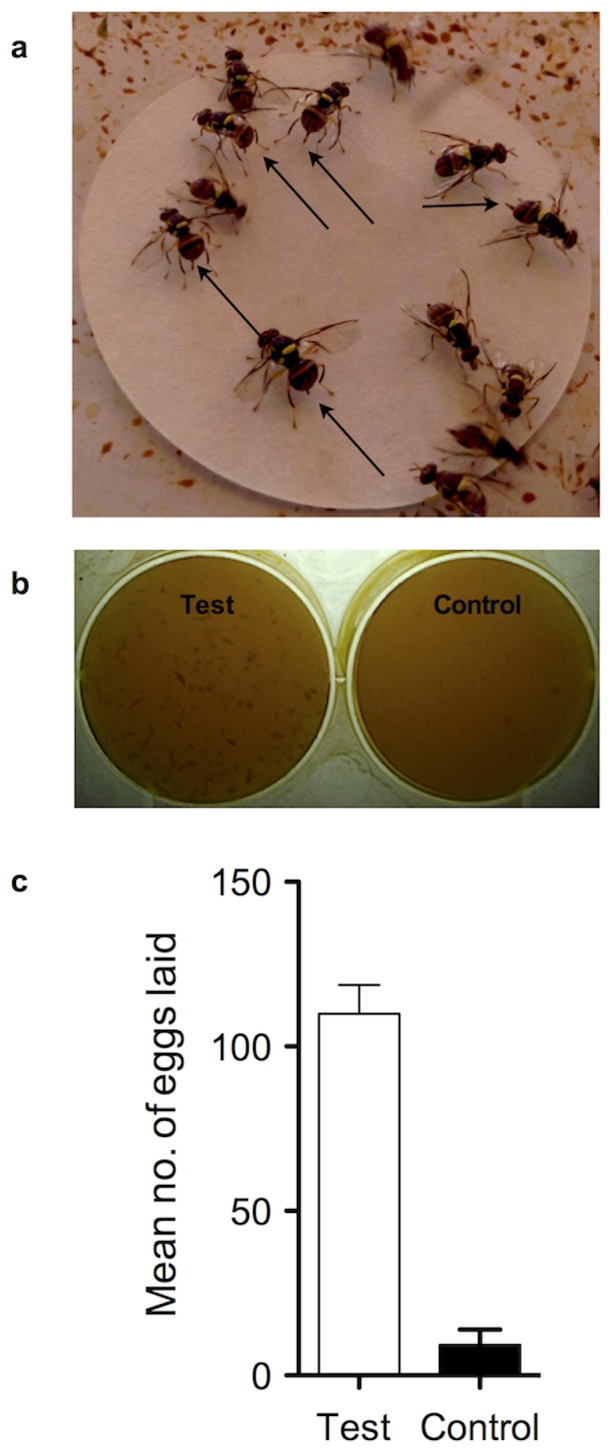
Behavior and egg-laying response of *B. dorsalis*. (a) Behaviour assay where *B. dorsalis* were presented with a filter paper disc containing γ-octalactone. Arrows show the hallmark oviposition behavior of extending the ovipositor and probing action (for better view watch video S1). (b & c) Egg-laying response of *B. dorsalis* to a pulp disc with γ-octalactone (Test) or without (Control) in a 24-h oviposition bioassay. More eggs were laid on the test disc (*P*<0.0001, one-tailed paired *t* test of non-transformed data). Error bars = Standard error of mean.

Electrophysiologically active headspace volatiles from mango cv. Alphonso, heptane, myrcene, (*Z*)-ocimene, (*E*)-ocimene, allo-ocimene, (*Z*)-myroxide and γ-octalactone, were tested [16]. These compounds were presented singly on filter paper discs (5 µL of 0.05 ppm; 50 mm disc diameter) to gravid female fruit flies. The oviposition behavior of flies in response to each of these potential cues was recorded. It was observed that only γ-octalactone triggered oviposition behavior (Fig. 1a & Video S1). None of the other compounds elicited this behavior. This behavior-assisted assay allowed us to identify a specific oviposition-stimulant from a range of electrophysiologically active compounds [16].

**Figure 2:**
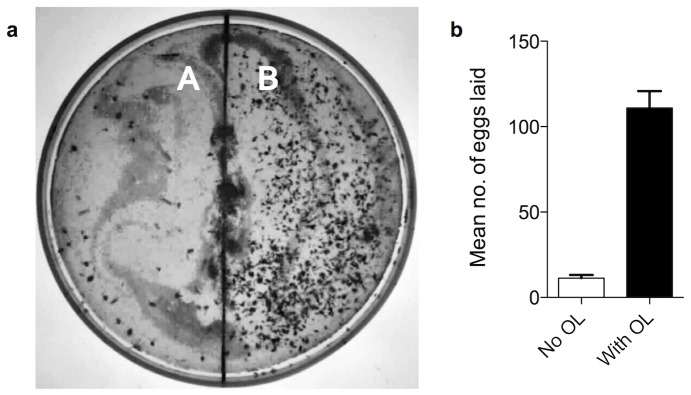
γ-Octalactone is an oviposition stimulant and an oviposition site recognition cue. (a) The clear discrimination of the untreated (No OL, γ-octalactone not present) half (A) and γ-octalactone treated (OL, γ-octalactone present) half (B) by *B. dorsalis* in a single plate two-choice assay. (b) Number of eggs laid into treated and untreated pulp (one-tailed paired *t* test, *n* = 30, *t = *11.27, *df* = 19, *P*<0.0001). Error bars = standard error of mean.

Next, we asked whether γ-octalactone aided oviposition site-selection. To evaluate this possibility a binary-choice oviposition assay was designed to evaluate the oviposition site preference of female *B. dorsalis*. Gravid *B. dorsalis* females were offered a choice of pulp discs with and without γ-octalactone. Gravid *B. dorsalis* females clearly differentiated between treated and untreated pulp discs (Fig. 1b) and directed significantly more, 95.85±8.7% (SEM), eggs into γ-octalactone treated than into untreated pulp discs (*t = *11.27, *df* = 19, *P*<0.0001) (Fig. 1c), indicating that γ-octalactone allowed the fly to select oviposition-sites. We then conducted an experiment with more complex volatile blends to test if γ-octalactone was the key cue mediating oviposition even when other host volatiles (a mixture of heptane, myrcene, (Z)-ocimene, (E)-ocimene, allo-ocimene, (Z)- and myroxide in the natural ratio but without γ-octalactone) were present. Single-plate two choice assay was particularly designed to tease apart attraction and oviposition behavior as well as to determine the behavioral importance of the ‘key’ compound, γ-octalactone. Gravid female flies were offered a pulp disc divided into two halves with one half containing natural ratios of electrophysiologically active volatiles devoid of γ-octalactone and the other half containing natural ratios of electrophysiologically active volatiles with γ-octalactone. Significantly more eggs (90.79±2.4%) were laid into the half containing γ-octalactone (*t* = 9.678, *df* = 11, *P*<0.0001) than into the other half devoid of γ-octalactone (Fig. 2a & 2b) indicating that γ-octalactone was responsible for mediating oviposition decision in female *B. dorsalis*.

Insect behaviors, such as oviposition site-selection, can be triggered by particular cues that occur within the context of the animal's environment for which there is an innate bias [14]. It is hypothesized that activity of neurons responding to these critical cues is typically consistent across a wide range of concentrations, which is termed concentration invariance [20]. In order to test for an innate bias towards γ-octalactone, we first carried out electrophysiological studies using the antenna of gravid females as evidence suggests that antenna plays a key role in perception of volatile compounds. Recordings were carried out by pulsing air from hexane on filter paper (control) or filter paper applied with different concentrations of γ-octalactone. Electroantennogram (EAG) responses (Fig. S2) were compared to the control. The fly's antenna responded equally to γ-octalactone concentrations ranging from 0.05–100 ppm and is evident through a dose-response curve (Table 1 & Fig. S1). One-way ANOVA of the mean response between the concentration ranges showed no significant difference (*n* = 6, *F* = 0.9469, *P* = 0.4746) between test concentrations between 0.05–100 ppm. Second, we conducted a choice bioassay where the flies (10 flies) were presented with pulp discs containing different concentrations (0.05–100 ppm) of γ-octalactone and were allowed to oviposit for a period of 24 h. The flies could not distinguish between the concentrations of γ-octalactone and directed equal number of eggs into pulp that were not significantly different at any given test concentrations of the cue (*n* = 6, *F* = 0.5584, *P* = 0.7594) (Table 1). These results suggest that the concentration of the cue did not influence the fly's oviposition response and that γ-octalactone was a “critical cue” used by *B. dorsalis* to distinguish suitable oviposition sites.

**Table 1:** EAG and ovipositional response of *Bactrocera dorsalis* to varying concentrations of γ-octalactone.

Concentration of γ-octalactone (ppm)	EAG response in mV ± SEM	Mean no. of eggs laid/female ± SEM
Control	0.096±0.05^a^	0.4±2.17^a^
0.05	0.582±0.11^b^	25.4±3.23^b^
0.1	0.514±0.06^b^	21.6±5.97^b^
0.5	0.458±0.10^b^	23.4±4.75^b^
1.0	0.497±0.03^b^	27.7±6.34^b^
10.0	0.660±0.15^b^	23.8±2.71^b^
50.0	0.693±0.17^b^	20.2±3.98^b^
100.0	0.681±0.15^b^	27.8±4.31^b^

Control = Hexane; SEM: Standard error of mean; Means within a column followed by the same letters ^a^ or ^b^ are not significantly different at *P*<0.001 (ANOVA); ppm: parts per million.

### Flies have developed an IRT tuned to γ-Octalactone

Whereas considerable attention has focused on the theoretical aspects of recognition templates, little is known about the actual cues triggering an IRT [2], [9], [21]. Our behavioral data suggest that gravid *B. dorsalis* females use γ-octalactone to recognize oviposition sites. To confirm the presence of an innate recognition template (IRT) tuned to γ-octalactone, we reared 3 groups of flies from a wild strain of *B. dorsalis.* Group 1 was reared exclusively on mango (*Magnifera indica* cv. Alphonso), group 2 on banana (*Musa sp*. cv. elakki bale) and group 3 on guava (*Psidium guajava* cv. Allahabad safeda). Thus, groups 2 and 3 were not exposed to mango cues until the experiment. Each group was reared for 3 generations and third generation flies were used for bioassays. GC/MS analysis of the headspace volatile of banana and guava indicated that γ-octalactone was not present. This was verified because we did not want olfactory imprinting of γ-octalactone to take place [22]. To examine oviposition-stimulants in the headspace volatiles of guava and banana, we subjected gravid flies from group 2 and 3 to behavior screening assay. Headspace volatiles (5 µL/filter paper disc) of banana and guava were presented to respective groups. It was observed that flies were attracted to the volatiles but did not show any oviposition behavior. This confirmed that guava or banana headspace volatile did not instigate oviposition behavior. Having confirmed this, we subjected naïve gravid female flies (30 flies; 15 days of age) from each of the three groups to behavioral screening assays.

**Figure 3:**
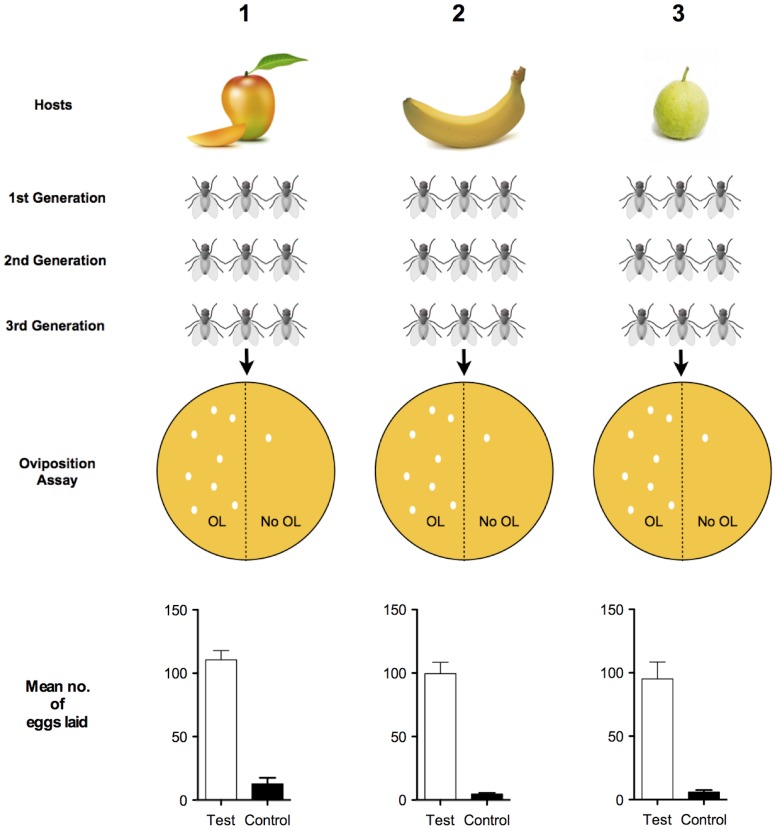
Schematic representation of the response of flies with different rearing histories towards γ-octalactone. *B. dorsalis* was reared on 3 different hosts. The final (third) generation gravid females were used in oviposition bioassays. A one-tailed *t* test revealed a significant difference (*P*<0.0001) between Test (OL, γ-octalactone present) and Control (No OL, γ-octalactone not present). One-way ANOVA between the tests of group 1 (mango), 2 (banana) and 3 (guava) showed no significant difference in the mean no. of eggs laid.

Flies from all groups showed oviposition behavior after exposure to γ-octalactone. In a choice assay, naïve gravid female flies that had no experience with γ-octalactone or oviposition site-selection were presented with pulp discs with and without γ-octalactone. The naïve gravid female flies with different rearing histories directed significantly more eggs into pulp discs containing γ-octalactone. Naïve gravid females from group 1, 2 and 3 directed 95.53±8.5% (*t* = 10.45, *df* = 19, *P*<0.0001); 94.16±7.5% (*t* = 6.659, *df* = 19, *P*<0.0001) and 89.55±7.1% (*t* = 12.09, *df* = 19, *P*<0.0001) eggs into pulp discs respectively. One-way ANOVA between the groups showed that the number of eggs directed by naïve females into pulp discs was not significantly different (*F* = 0.6159, *P* = 0.5437) (Fig. 3). Although naïve *B. dorsalis* females did not have any previous experience or exposure to γ-octalactone, they were able to detect the presence of the cue and direct the majority of their eggs into γ-octalactone treated pulp discs.

### Gravid flies show hardwired preference for oviposition sites with γ-octalactone

**Figure 4:**
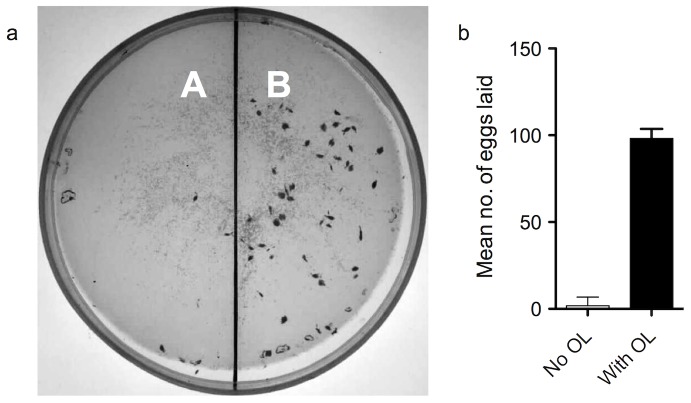
γ-Octalactone is a crucial cue that elicits oviposition in *B. dorsalis*. (a) Flies were allowed to oviposit into an empty agar disc. Area A contained all EAD active mango cues (cv. Alphonso) at natural ratios except γ-octalactone; Area B contained γ-octalactone alone. Flies could clearly distinguish between the untreated area (A) and the γ-octalactone treated area (B) (One-tailed *t* test, *t* = 6.610, *df* = 8, *P*<0.0001). (b) The mean number of eggs laid into A or B. Oviposition in *B. dorsalis* was mediated by γ-octalactone. Error bars = Standard error of mean.

Because insects demonstrate a hard-wired response to critical cues, it is possible that insects can be tricked by critical cues even if they are outside their usual context [11]. To demonstrate that γ-octalactone induces a hardwired response and tricks gravid *B. dorsalis* females, flies were presented with a choice between empty agarose discs with one half containing γ-octalactone and the other half disc without γ-octalactone. The results were surprising as the flies laid 98.13±5.6% of eggs (*t* = 7.357, *df* = 8, *P*<0.0001) into empty agarose disc with γ-octalactone (Fig. 4). This outcome confirmed that γ-octalactone was enough to induce oviposition and the ovipositional response is channeled through an IRT in the olfactory pathway overriding inputs from other modalities. To provide further confirmation, gravid female flies were presented with non-hosts (cucumber and potato) treated with γ-octalactone and control non-hosts without γ-octalactone. The flies laid 100% of their eggs into the treated non-hosts and no eggs were recorded in untreated non-hosts. These results show that the IRT is strongly tuned to γ-octalactone and this compound mediates the oviposition response in the Oriental fruit fly, *B. dorsalis*.

## Discussion and Conclusion

The placement of eggs by gravid females is a crucial step that decides the fitness of *B. dorsalis* larvae [23]. Though, females can readily discriminate between oviposition sites of different quality, a trade-off exists between selecting sites where larval development rates are maximised and quick allocation of eggs in the time available with competition for resources from conspecifics [24]–[26]. Therefore, it is beneficial for flies to develop an IRT for specific cues that allow them to rapidly discern suitable oviposition sites. Our results show that γ-octalactone is a crucial cue for *B. dorsalis* oviposition and it appears to have developed an IRT tuned to this compound during the course of evolution. Indeed, in our experiments, flies laid eggs even into unfavorable oviposition sites and non-hosts when γ-octalactone was present.

Presumably in natural ecological situations γ-octalactone only occurs where there are suitable fruits for oviposition such as in the preferred host, mango. Although γ-octalactone elicits oviposition behavior, flies also lay eggs into guava and banana that are devoid of this cue. Being a polyphagous herbivore, it can adapt to different hosts depending on their availability in the habitat and there could be alternative cues that elicit oviposition in other host fruits. Plasticity of ovipositional preference is undoubtedly critical among polyphagous species whose choices of hosts vary in availability unpredictably. Both evolutionary relationships and associative learning play important roles in allowing gravid females to make rapid decisions that maximize their fitness [27]–[29].

We asked what the evolutionary reason might be for the fly's strong, innate, oviposition response to γ-octalactone. Lactones are strong anti-fungal agents inhibiting growth of molds even at small concentrations and their presence means that the mango is ripe and contains less terpene compounds [15], [30]. Therefore, the innate oviposition behavior may be due to a recognition template tuned to γ-octalactone that alerts flies not only to the presence of an appropriate oviposition site but also to a clean, healthy nursery devoid of fungal saprophytes and terpenes that can harm the development of the fly's offspring.

Here we show that, γ-octalactone, present in mango, can activate an IRT tuned for oviposition. γ-octalactone alone is both necessary in mango odors and sufficient to instigate oviposition, overriding other exploratory behaviors in *B. dorsalis*. Previous studies have reported similar recognition templates activated by specific chemical cues [1], [3], [13], but were acquired through learning. Our study illustrates that an oviposition site recognition template acquired through evolution of the fly with its host has become an innate recognition template that is passed on through generations. The identification of critical cues and their recognition templates will increase our understanding of the mechanisms underpinning evolved innate behaviors and allow future studies of the dedicated neural circuits underlying these innate behaviors [31]–[33].

## Supporting Information

Figure S1 Dose-response curve of gravid *B. dorsalis* antenna to γ-octalactone at varying concentrations ranging from 0.005–100 ppm.
(EPS)

Figure S2 EAG response profiles of gravid female *B. dorsalis* to different concentrations (in ppm) of γ-octalactone (hexane was used as control). Six replicates per concentration were done. A single EAG peak per concentration is shown.
(PDF)

Video S1 Oviposition behavior of the oriental fruit fly in response to γ-octalactone on a filter paper disc.
(MP4)
