# Supplementary figures and images for: Oviposition Site-Selection by Bactrocera dorsalis Is Mediated through an Innate Recognition Template Tuned to γ-Octalactone
Source: PLoS One. 2014 Jan 23;9(1):e85764. doi: 10.1371/journal.pone.0085764 (PMC3900432; doi:10.1371/journal.pone.0085764)

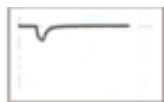

0.005

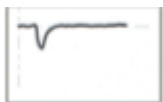

0.01

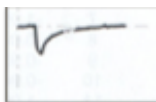

0.05

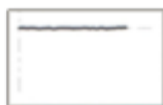

Control (Hexane)

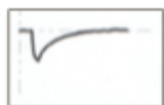

0.1

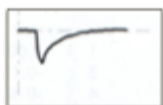

0.5

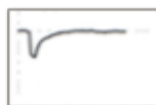

1

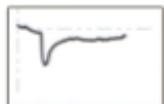

10

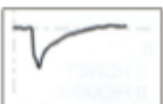

50

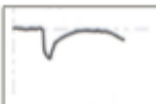

100

Supplement: Figure S2 — EAG response profiles of gravid female B. dorsalis to different concentrations (in ppm) of γ-octalactone (hexane was used as control). Six replicates per concentration were done. A single EAG peak per concentration is shown. (PDF) [file pone.0085764.s002.pdf]
